# Do predators have a role to play in wetland ecosystem functioning? An experimental study in New England salt marshes

**DOI:** 10.1002/ece3.7880

**Published:** 2021-07-21

**Authors:** Alexandria C. Moore, Oswald J. Schmitz

**Affiliations:** ^1^ School of the Environment Yale University New Haven USA

**Keywords:** bottom‐up control, consumers, ecosystem functions, predators, salt marsh, top‐down control, wetlands

## Abstract

The historical ecological paradigm of wetland ecosystems emphasized the role of physical or “bottom‐up” factors in maintaining functions and services. However, recent studies have shown that the loss of predators in coastal salt marshes can lead to a significant reduction in wetland extent due to overgrazing of vegetation by herbivores. Such studies indicate that consumers or “top‐down” factors may play a much larger role in the maintenance of wetland ecosystems than was previously thought. The objective of this study was to evaluate whether altering top‐down control by manipulating the presence of predators can lead to measurable changes in salt marsh ecosystem properties. Between May and August of 2015 and 2016, we established exclosure and enclosure cages within three New England coastal wetland areas and manipulated the presence of green crab predators to assess how they and their fiddler and purple marsh crab prey affect changes in ecosystem properties. Predator presence was associated with changes in soil nitrogen and aboveground biomass at two of the three field sites, though the magnitude and direction of these effects varied from site to site. Further, path analysis results indicate that across field sites, a combination of bottom‐up and top‐down factors influenced changes in measured variables. These results challenge the growing consensus that consumers have strong effects, indicating instead that predator impacts may be highly context‐dependent.

## Graphical abstract

The objective of this study was to evaluate whether altering top‐down control by manipulating the presence of predators can lead to measurable changes in salt marsh ecosystem properties. Predator presence was associated with changes in soil nitrogen and aboveground biomass at two of the three field sites, though the magnitude and direction of these effects varied from site to site. These results challenge the growing consensus that consumers have strong effects, indicating instead that predator impacts may be highly context‐dependent.

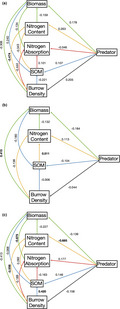

## 1 INTRODUCTION

From freshwater to saltwater, marshes to mangroves, wetlands are diverse ecosystems of critical social, cultural, economic, and environmental importance (Costanza et al., 1997; Constanza et al., 2017; Moreno‐Mateos et al., 2012). The services and benefits they provide include food production, habitat refugia, and disturbance regulation, valued in the trillions of dollars annually (Costanza et al., 2017; Zedler, 2000). Despite their importance, various anthropogenic activities such as habitat destruction, overfishing, and larger climate‐driven events have resulted in large‐scale losses of wetlands and their associated ecosystem functions and services (Kirwan & Megonigal, 2013; Lotze et al., 2006; Myers et al., 2007; Paerl et al., 2001). Considerable attention has therefore been devoted to restoring these habitats to recover lost functions and safeguard them into the future (Meli et al., 2014; Moreno‐Mateos et al., 2012). Yet, with few exceptions, restored wetlands exhibit reduced biological structure and biogeochemical function compared with natural reference wetlands (Meli et al., 2014; Moreno‐Mateos et al., 2012, 2017; Salvesen, 1994; Zedler & Callaway, 1999).

Most wetland restoration initiatives are based on the historical notion that wetland structure and function are predominantly controlled from the bottom‐up by plant–soil interactions (Odum & Smalley, 1959; Sala et al., 2008; Smalley, 1960; Teal, 1962). It follows that by building up plant communities, restoration will lead to habitats that facilitate the natural reestablishment of herbivore and predator species populations along with associated ecosystem functions. However, research over the last decade has increasingly shown that once established, herbivores and predators may play instrumental roles in facilitating wetland functioning through top‐down interactions that significantly impact the plant community and soil conditions (Altieri et al., 2012; Bertness et al., 2008, 2014; Bertness, Brisson, Coverdale, et al., 2014; Moore, 2018; Renzi et al., 2019; Silliman & Bertness, 2002). For instance, in the Netherlands, livestock grazing is commonly used as a wetland management tool that, in some cases, can increase soil bulk density, reducing redox potential and nutrient mineralization rates, and enhancing carbon storage in marsh sediments (Bakker, 1989; Elschot et al., 2013; Kiehl et al., 1996). Furthermore, Bertness, Brisson, Coverdale, et al. (2014) found that in the absence of predators, herbivorous crab densities within a Massachusetts salt marsh significantly increased, leading to a substantial reduction in smooth cordgrass biomass with a concomitant loss of stored sediment carbon.

These and other studies challenge the commonly held view that wetland ecosystems are largely bottom‐up controlled and therefore suggest the importance of considering the multiple and potentially interactive outcomes of bottom‐up and top‐down effects and feedbacks (Altieri et al., 2012; Bertness, Brisson, Coverdale et al., 2014; Coverdale et al., 2013, 2014). Under a bottom‐up control worldview, the biotic diversity of marshes and related trophic interactions are treated as measures of ecosystem health as opposed to a top‐down view that would treat them as factors that may influence ecosystem health (Kentula, 2000). However, the idea that top‐down processes may influence salt marsh ecosystem structure and function with implications for conservation and restoration has not been tested widely and is thus in need of significant empirical evaluation. The objective of this study was to evaluate whether altering top‐down control by manipulating the presence of a predator can lead to measurable changes in salt marsh ecosystem processes and to compare these changes to those due to bottom‐up factors. This study aims to build on previous work showing context‐dependent consumer control in New England coastal salt marshes (Moore, 2018) by conducting a repeated‐measures experiment over a 2‐year period and evaluating both top‐down and bottom‐up effects. As such, this study contributes to a growing body of literature evaluating the relative importance of bottom‐up and top‐down controls in restoring ecosystems (Fraser et al., 2015).

## 2 METHODS

### 2.1 Study sites

Field experiments were conducted in three locations of similar tidal salt marsh situated along 32 km of the Connecticut coastline: Farm River State Park in East Haven, CT, USA (41°15'21.82"N, 72°51'24.12"W), Fence Creek in Madison, CT, USA (41°16'33.25"N, 72°35'10.24"W), and Hammonasset Beach State Park in Madison, CT, USA (41°15'59.88"N, 72°33'30.30"W). These sites were selected because they exhibited evidence of reduced predator populations and a similar tidal regime as indicated in our previous study at these locations (Moore, 2018).

### 2.2 Study system

Tidal salt marsh communities along the New England coastline are dominated by salt‐tolerant grasses, such as smooth cordgrass and saltmeadow cordgrass (*Spartina alterniflora* and *Spartina patens*, respectively). They also contain several detritivore, herbivore, and predator species, of which fiddler crabs (*Uca pugnax* and *Uca pugilator*) and purple marsh crabs (*Sesarma reticulatum*) may have a dominant influence in maintaining salt marsh properties and functions (Bertness, 1992; Moore, 2019). The fiddler crab is a detritivore that alters the landscape through its burrowing behavior and deposit feeding, while the purple marsh crab is a burrowing herbivore that directly consumes marsh vegetation aboveground and belowground (Bertness, 1985; Holdredge et al., 2009; Miller, 1961). Together, the natural behaviors of these two species may contribute to the maintenance of processes and properties within salt marsh ecosystems (Moore, 2019). In particular, fiddler crabs may decrease soil organic matter content, increase soil inorganic nitrogen availability, increase the rate of soil nitrogen absorption (i.e., a measure of the relative availability of soil nitrogen), and increase decomposition by sifting through the sediment for food and continuously turning over the soil by maintaining burrows. Primary production may then be positively impacted by fiddler crab detritivory and burrowing behavior as a result of improved soil conditions (Bertness, 1985; Gribsholt et al., 2003; Penha‐Lopes et al., 2009; Smith et al., 2009). Alternatively, purple marsh crabs may directly decrease aboveground biomass through herbivory, leading to associated reductions in plant material entering the detrital chain in the form of soil organic matter, while simultaneously improving soil conditions through burrowing behavior (Bertness, Brisson, Coverdale, et al., 2014; Coverdale et al., 2012; Penha‐Lopes et al., 2009; Smith et al., 2009).

Fiddler crabs and purple marsh crabs are also important prey of the European green crab (*Carcinas maenas*, hereafter “green crab”), a non‐native opportunistic predator found along the New England coast (Coverdale et al., 2013; Leignel et al., 2014). The green crab is a nonburrowing, but highly adaptable species that can be found foraging across the salt marsh landscape throughout the tidal regime and hiding in other species’ burrows at low tide to avoid predation and prevent desiccation (Bertness & Coverdale, 2013). The presence or absence of the green crab may moderate the effects that its prey have on ecosystem processes (Table 1). Specifically, green crabs should indirectly facilitate an increase in soil organic matter content, a decrease in soil inorganic nitrogen content and the rate of soil nitrogen absorption, and an increase aboveground biomass production. Altogether, these species comprise the ecological community of focus in the present study.

**TABLE 1:** Predicted impact of predator presence or absence on measured variables. The positive symbol indicates an increase in the measured variable relative to control conditions while the negative symbol indicates a decrease in the measured variable relative to control conditions

	Burrow Density	Soil Organic Matter	Nitrogen Content / Absorption	Aboveground Biomass
Predator	‐	‐	‐	+
No Predator	+	‐	+	‐

### 2.3 Manipulation experiment

Field experiments were conducted from May to August in 2015 and 2016. This time period covers the *S*. *alterniflora* and *S. patens* growing season when each target consumer species was most active (Bertness, 1991, 1992). Within each study site, experimental blocks comprising groups of three plots were established and randomly placed along the creek‐bank edge of the low marsh where smooth cordgrass was the dominant vegetation. The three plots consisted of two manipulation plots and one control plot, which represented existing conditions in the field site. Our previous study within these sites demonstrated that an additional cage control plot was not needed because there were no cage artifacts on response variables (Moore, 2018). Each site contained six blocks of three plots except for Hammonasset Beach State Park, where treatments could only be replicated in five blocks due to space limitations. Such replication is consistent with previous experiments testing for trophic control of salt marsh ecosystems (Bertness, Brisson, Coverdale, et al., 2014; Silliman et al., 2004). Experimental methods are described in additional detail in Moore (2018).

Manipulation plots were assigned to one of two treatments: (a) Predator Absent and (b) Predator Present. The Predator Absent plots fully excluded the green crab from accessing the cages. The Predator Present plots were stocked at an average field density of three individuals per cage (Bertness & Coverdale, 2013; Gregory & Quijon, 2011) using green crab individuals purchased from local bait and tackle shops in June 2015 and 2016. Green crabs were purchased, rather than trapped on site, to ensure that experimental cages were stocked at the same time. Individuals used for stocking were all adults with a carapace width ranging from 50mm to 75mm and were not individually sexed. Experimental cages were checked weekly and restocked with crabs of similar size to replace individuals that died over the course of the experiment. Within each of the manipulation and control plots, three holes created from initial soil samples were augmented to a diameter of 10cm and a depth of 20cm using a hand trowel to provide green crabs with burrows to serve as refuges from desiccation and predation during low tide.

### 2.4 Measurements and laboratory analyses

All measurements except aboveground biomass were taken prior to the onset of the experiment in May of each year to determine initial conditions. Measurements were made again at the end of the experiment in August of each year to evaluate treatment effects relative to initial conditions.

Burrow density was used to estimate population density of fiddler and purple marsh crabs. This standard measure is widely used as a proxy for the number of individuals within a given area (Coverdale et al., 2014; Gittman & Keller, 2013). Soil organic matter (SOM) content was determined using the loss‐on‐ignition method (Nelson & Sommers, 1996). In 2015, soil inorganic nitrogen concentration was measured using a potassium chloride extraction method (Robertson et al., 1999; Robertson, Wedin, et al., 1999). These methods were conducted as previously described in Moore, 2018.

In 2016, the rate of soil nitrogen absorption was measured instead of soil nitrogen concentration in order to more directly evaluate the impact of experimental treatments on soil processes integrated over the growing season rather than making inferences based on sampling at fixed points in time. The rate of soil nitrogen absorption was measured in situ pre‐ and postexperimental period using ion exchange resin strips (Qian & Schoenau, 1996). Anion and cation exchange membranes (General Electricals) were cut from a bulk sheet down to 2.5 x 10cm strips and differentiated using a hole punch. On one end of each strip, bright pink zip‐ties were affixed through the hole punch to act as a clear visual identifier once placed in the field. To prepare strips for use, they were regenerated using an acid wash to remove any existing nutrient ions (Qian & Schoenau, 1996). Once regenerated, strips were rinsed with deionized water and placed into anion‐ and cation‐separated, labeled, and clean Ziploc bags until placed in the field. At each field site, two anion and two cation strips were placed into each plot. Field methods and laboratory analyses were then completed following procedures described in Moore (2019). Total nitrogen absorbed (µg/cm^2^/day) was calculated using the following formula:

$$\left[\left(N\;Concentration\;in\frac{\mu g}{\text{ml}}\right)\times 70\;\text{ml}\;of\;KCl\right]÷\left[50.8\;{\text{cm}}^{2}strip\;area÷8\;days\;in\;ground\right]$$

Aboveground biomass was evaluated at the end of the growing season in August of both experimental years. All standing vegetation, including *S*. *alterniflora*, *S. patens*, and other species present within each plot, was collected by cutting plants at the ground level using garden shears. Vegetation was placed in labeled paper bags and transported to Yale laboratory facilities. Bags were left out to air dry for several weeks and then weighed using a top‐loading scale to 0.1 accuracy. Since biomass samples were air‐dried, these measurements reflect relative biomass rather than absolute biomass.

### 2.5 Data analysis

All statistical analyses were carried out using R Studio (v. 1.4.1106). The relative changes in burrow density, SOM content, soil nitrogen concentration, and rate of soil nitrogen absorption were calculated for both years where applicable. Relative change was defined as the difference between the initial and final conditions within a given year divided by the initial conditions of that same year. For variables measured in both experimental years, the data from both years were combined into one dataset and statistically analyzed together. For aboveground biomass, measurements taken at the end of the experimental period for both years were used in all statistical analyses.

The relative change in burrow density, SOM content, soil nitrogen concentration, and rate of soil nitrogen absorption were analyzed using generalized linear mixed‐effects models (GLMM) while final aboveground biomass measurements were analyzed using a linear mixed model (LMM). Treatment, site, and year (where applicable) were set as fixed effects for each model with block (i.e., treatment groupings) set as a random effect nested within site. This nesting allowed us to address any potential autocorrelation arising from nonindependence among treatment groups. Models also included treatment‐by‐site and treatment‐by‐year interaction terms to identify site‐ or year‐specific differences. For the GLMMs constructed for SOM content, soil nitrogen concentration, and rate of soil nitrogen absorption, the error distributions were evaluated using the “fitdistrplus” library. Akaike's information criterion (AIC) scores and the fit of the data were used to select the best model for each response variable. Each GLMM model was fit to a gamma error distribution using a log link. For response variables that produced significant interaction effects between treatment and site or treatment and year, additional models for individual sites and years were constructed to determine context‐dependency of treatment effects. For these, the aforementioned fixed and random effects were used but site or year was removed from the model. Models were analyzed using the “lme4” library (Bates et al., 2015) along with the “lmerTest” (Kuznetsova et al., 2017) and “multcomp” libraries (Hothorn et al., 2008) to get significance estimates. When significant relationships occurred, Tukey contrasts were performed to determine which means were significantly different between experimental treatments. Each response variable was also analyzed using an analysis of variance (ANOVA) to test for differences between study sites.

Mixed models and ANOVAs provide key insights into how experimental treatments and additional effects may influence measured variables. However, observed changes in multiple response variables may be correlated with one another or cause feedbacks that swamp out main direct effects. Therefore, a path analysis was used to account for variation and covariation among each of the response variables (McMahon et al., 2012) and to evaluate the degree to which measured ecosystem responses were controlled by bottom‐up or top‐down effects. The purpose was not to construct multiple models to evaluate different hypotheses of causality; rather, the purpose here was to build a model including all relevant data in order to observe the relative contributions of each variable to measured responses. Path analyses models were constructed with data from all sites combined as well as for each individual site. Each model included terms for the relative change in burrow density, SOM content, soil nitrogen concentration, and the rate of soil nitrogen absorption; a term for the final aboveground biomass measurements; and a term representing the presence or absence of the predator consumer (“Predator,” coded as a categorical variable). For the path analysis model constructed for Fence Creek, data on the rate of soil nitrogen absorption were not included due to the lack of model convergence arising from missing values in the dataset. All models were constructed based on the predicted relationships between each of the response variables, resulting in the following structure:

Model≤Biomass∼Predator+BurrowDensity+Nitrogen+NAbsorption;Nitrogen∼Predator+BurrowDensity+OrganicMatter;NAbsorption∼Predator+BurrowDensity+OrganicMatter;OrganicMatter∼Predator+BurrowDensity+Biomass;BurrowDensity∼Predator

Path analyses were conducted using the “OpenMX” and “lavaan” libraries, and path diagrams were initially created using the “semPlot” library, then simplified using Microsoft Powerpoint (v. 16.50).

## 3 RESULTS

### 3.1 Mixed‐effects models

Mixed‐effects models indicated that the presence of predators was not a significant predictor of any response variable except the change in burrow density, where the Predator Absent treatment exhibited a significant decline in burrow density relative to the control (*p* =.03). The change in burrow density did not significantly differ between the Predator Absent and Predator Present treatment or between the control and Predator Present treatments. Although no overall predator effects were observed across all sites, there were significant treatment‐by‐site effects at several variables.

**FIGURE 1:**
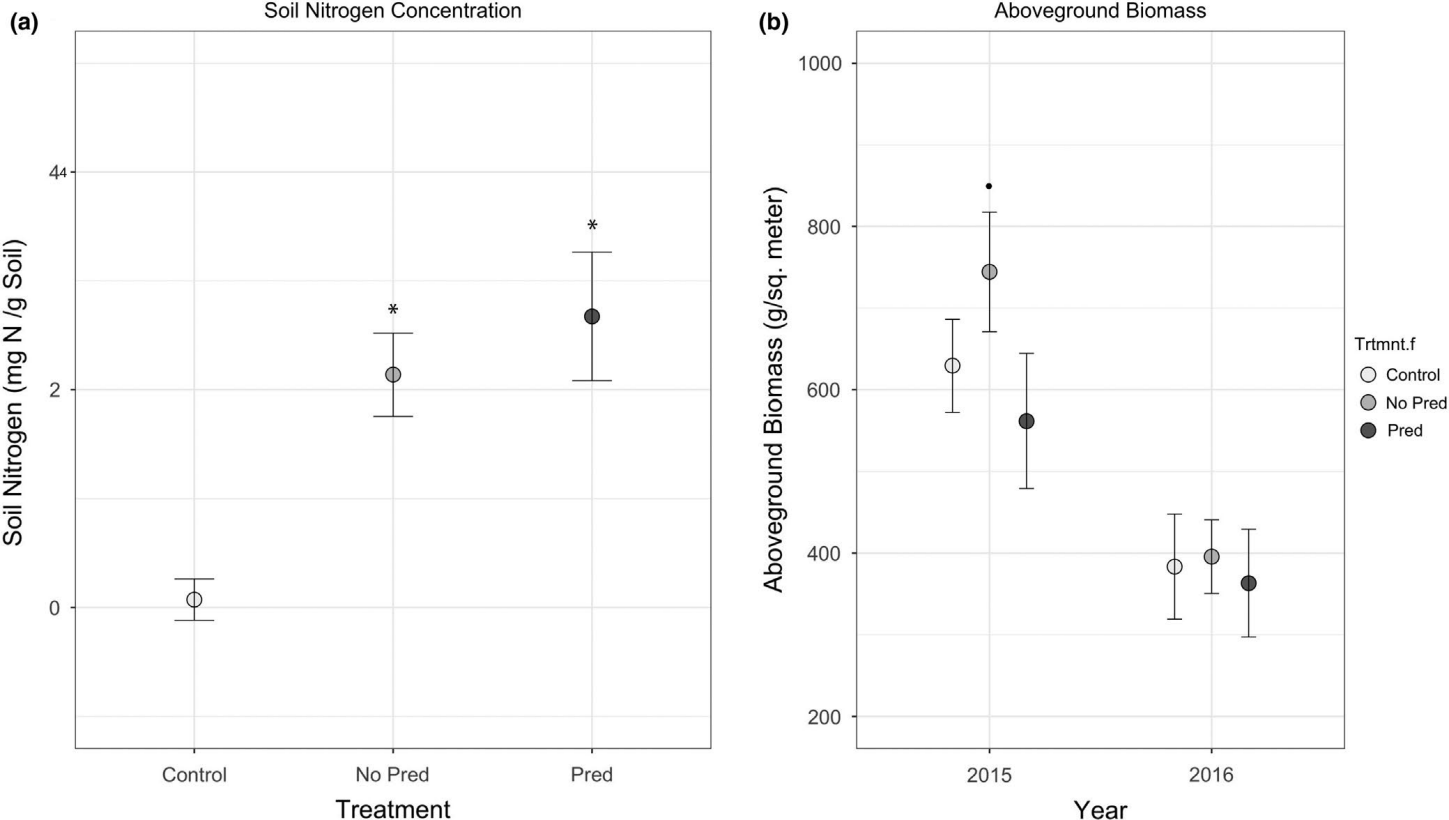
(a) Relative change in soil nitrogen content in 2015 and (b) final aboveground biomass measurements at Fence Creek in 2015 and 2016. Bars represent standard error, an asterisk represents significant difference compared with the control, and a black dot represents significant difference compared with experimental treatment (*p* <.05)

**FIGURE 2:**
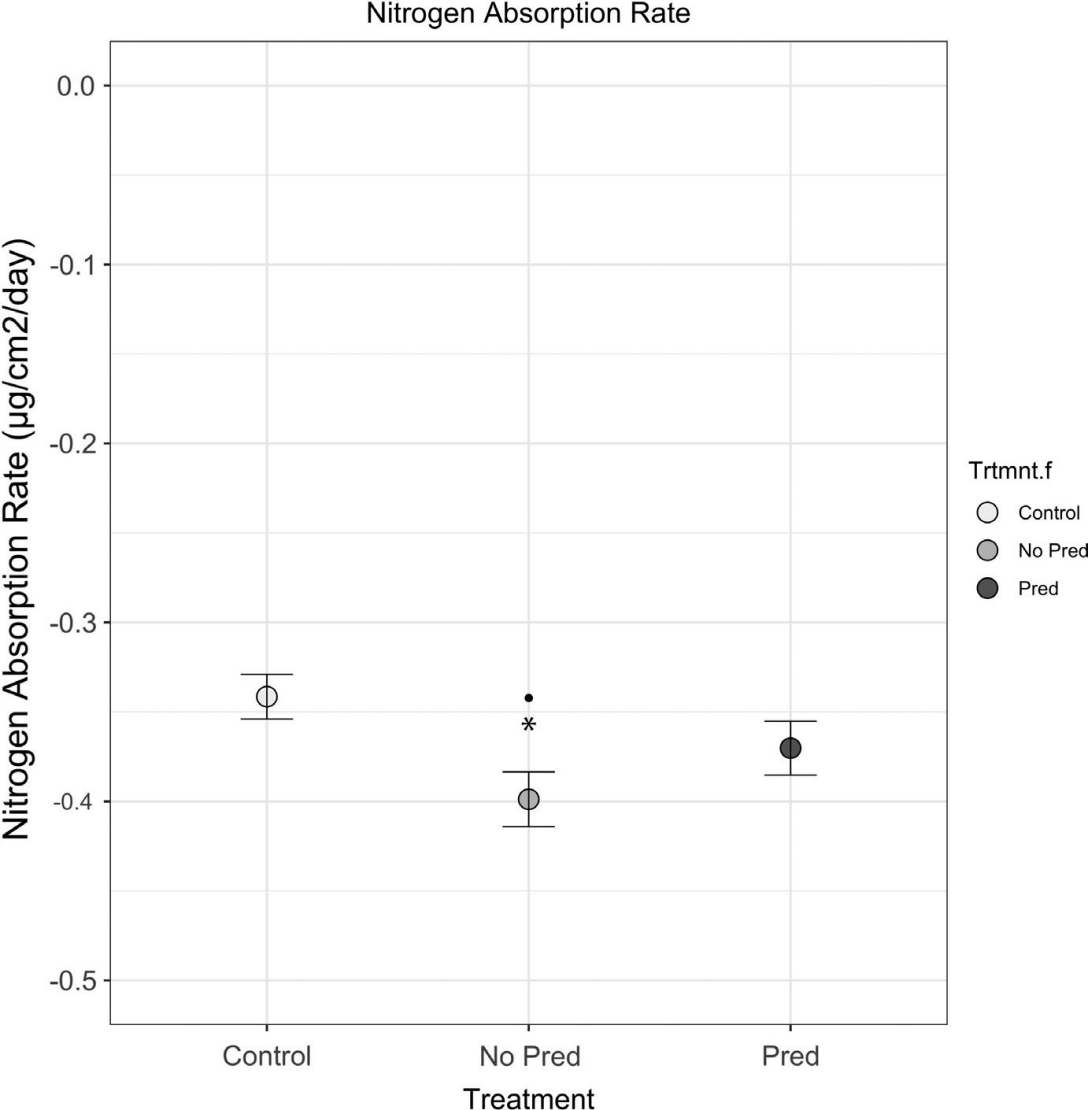
Relative change in the rate of nitrogen absorption in 2016 at Hammonasset Beach State Park. Bars represent standard error, an asterisk represents significant difference compared with the control, and a black dot represents significant difference compared with experimental treatment (*p* <.05)

At Farm River State Park, there was a significant decline in burrow density in the Predator Absent treatment compared with the control (*p* =.011) but there were no other significant differences between the remaining treatment combinations. At Fence Creek, there was a significant increase in soil nitrogen concentration in the Predator Absent treatment compared with the control (*p* <.0001) and in the Predator Present treatment compared with the control (*p* <.0001) (Table 2, Figure 1a). There was no difference in the change in soil nitrogen concentration between the two experimental treatments (*p* >.8). At this site, there was also significantly more aboveground biomass in the Predator Absent treatment compared with the Predator Present treatment (*p* =.038) (Table 2, Figure 1b). Aboveground biomass did not differ between the experimental treatments and the control here (*p* >.1). Finally, at Hammonasset Beach State Park, there was a significant decline in the rate of soil nitrogen absorption in the Predator Absent treatment compared with the control (*p* =.0086) and a marginal decline in the Predator Absent treatment compared with the Predator Present treatment (*p* =.054) (Table 3, Figure 2). There was no difference in the rate of soil nitrogen absorption between the Predator Present treatment and the control (*p* >.7) (Table 3, Figure 2).

**TABLE 2:** Mixed‐effects model results for soil nitrogen concentration and aboveground biomass across experimental treatments at Fence Creek. Bold values indicate significance at *p* <.05

	Soil Nitrogen Content				Aboveground Biomass			
Linear Hypotheses	Estimate	Std. error	z value	P	Estimate	Std. error	z value	P
Predator Absent ‐ Control	1.166	0.269	4.33	**4.23e−05**	144.0	78.8	1.83	0.160
Predator Present ‐ Control	1.305	0.271	4.82	**< 1e−05**	−38.5	78.8	−0.49	0.877
Predator Present – Predator Absent	0.138	0.267	0.52	0.863	−182.5	74.6	−2.45	**0.038**

**TABLE 3:** Mixed‐effects model results for the rate of soil nitrogen absorption across experimental treatments at Hammonasset Beach State Park. Bold values indicate significance at *p* <.05 while underlined values indicate marginal nonsignificance at *p* <.1

Linear Hypotheses	Estimate	Std. Error	z value	P
Predator Absent – Control	0.35974	0.12142	2.963	**0.0086**
Predator Present – Control	0.07919	0.12008	0.659	0.7870
Predator Present – Predator Absent	−0.28055	0.12141	−2.311	0.0542

In addition to experimental treatment effects, ANOVA results highlight significant site and year effects on several variables. The change in soil nitrogen concentration (*F*_(2,47)_ = 14.46, *p* <.0001) was significantly higher at Fence Creek relative to both Farm River State Park (*p* <.001) and Hammonasset Beach State Park (*p* <.0001). Further, the rate of soil nitrogen absorption (*F*_(2,44)_ = 8.373, *p* <.001) increased significantly at Farm River State Park compared with Fence Creek (*p* <.0001) and Hammonasset Beach State Park (*p* <.03). Finally, SOM content (*F*_(2, 78)_ = 5.355, *p* <.01) was significantly lower at Farm River State Park compared with both Fence Creek (*p* <.04) and Hammonasset Beach State Park (*p* <.02). Additionally, there was significantly less aboveground biomass measured across all three field sites and experimental treatments in 2016 relative to 2015 (*F*_(1,94)_ = 40.46, *p* <.0001).

### 3.2 Path analysis

**FIGURE 3:**
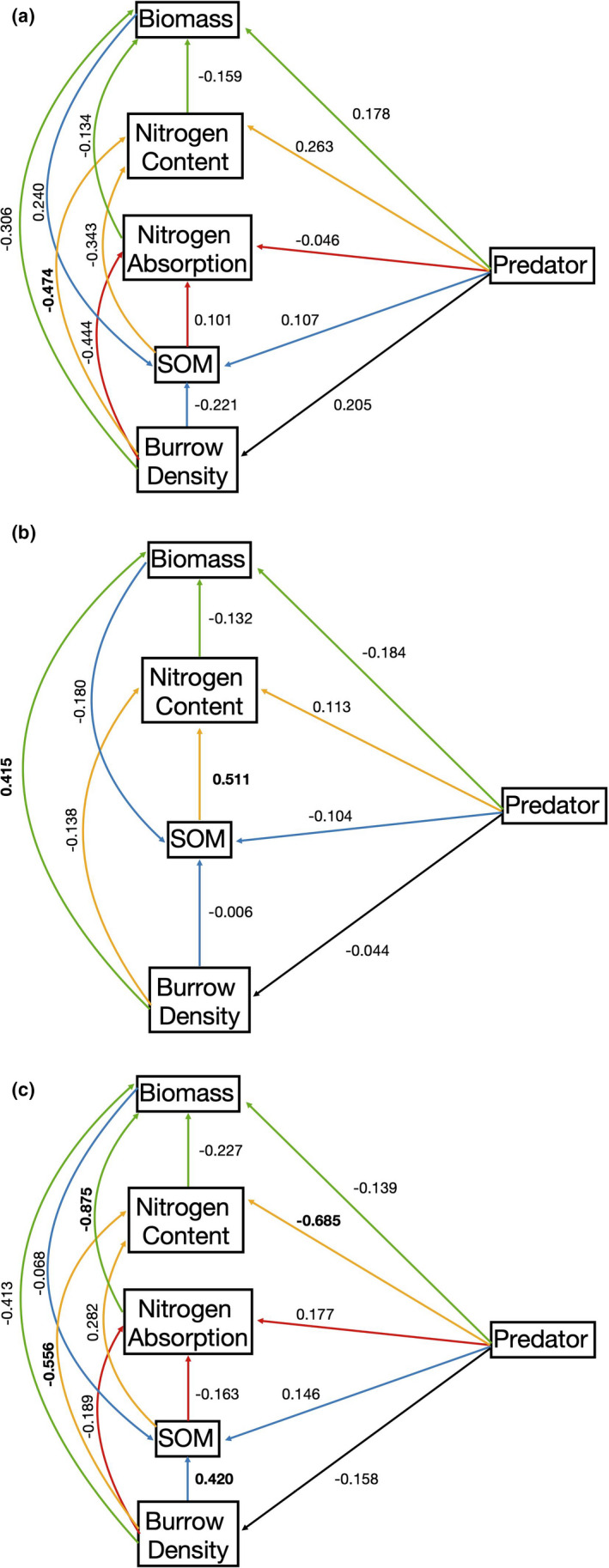
Results of a path analysis evaluating the effects on response variables at (a) Farm River State Park, (b) Fence Creek, and (c) Hammonasset Beach State Park. In the interaction web, line color corresponds with effects on response variables: black for burrow density, blue for soil organic matter content, orange for soil nitrogen content, red for the rate of nitrogen absorption, and green for biomass. Numbers next to the adjacent arrows are the path coefficients. Bold values indicate significance at *p* <.05

Across all field sites, standardized path analysis coefficients indicate that neither bottom‐up nor top‐down factors dominated in driving experimental outcomes except for the rate of soil nitrogen absorption, which significantly influenced aboveground biomass (Table 4). There were, however, significant bottom‐up and top‐down effects that differed among field sites (Figure 3a‐c). At Farm River State Park, the change in burrow density significantly influenced the change in soil nitrogen concentration (a top‐down effect); at Fence Creek, the change in SOM content significantly influenced the change in soil nitrogen concentration and the change in burrow density influenced aboveground biomass (a mixture of top‐down and bottom‐up effects); and at Hammonasset Beach State Park, the rate of soil nitrogen absorption influenced aboveground biomass, predator presence and the change in burrow density influenced the change in soil nitrogen concentration, and the change in burrow density influenced the change in SOM content (a mixture of top‐down and bottom‐up effects).

**TABLE 4:** Path analysis results for all sites combined. Bold values indicate significance at *p* <.05 while underlined values indicate marginal nonsignificance at *p* <.1

Regressions	Estimate	Std. Error	z value	P	Std. Coefficients
Biomass ~ Predator Burrow Nitrogen *N* Absorp.					
	−0.150	0.299	−0.501	0.616	−0.049
	0.121	0.479	0.253	0.800	0.034
	−0.007	0.249	−0.029	0.977	−0.004
	−27.454	6.671	−4.115	**0.000**	−0.554
Nitrogen ~ Predator Burrow OM					
	−0.042	0.240	−0.177	0.859	−0.023
	−0.307	0.253	−1.216	0.224	−0.143
	2.586	1.358	1.905	0.057	0.354
*N* Absorp. ~ Predator Burrow OM					
	0.002	0.008	0.289	0.772	0.039
	0.001	0.014	0.045	0.964	0.009
	0.002	0.047	0.036	0.971	0.007
OM ~ Predator Burrow Biomass					
	0.018	0.027	0.660	0.509	0.072
	−0.021	0.036	−0.578	0.563	−0.071
	0.016	0.014	1.216	0.224	0.201
Burrow ~ Predator					
	0.056	0.090	0.617	0.537	0.065

## 4 DISCUSSION

### 4.1 Overview

Contrary to recent studies, the mixed‐effects models and path analysis results shown here indicate that strong top‐down control by predators was not ubiquitous among the salt marsh sites. Rather, a mixture of bottom‐up and top‐down factors variably influence changes in wetland ecosystem properties. These results contrast stated predictions about how top‐down control should impact variables measured across all field sites (cf. Table 1 and Table 5) and differ from previous findings of exclusive top‐down control in similar marsh systems along the Eastern US coast (Altieri et al., 2012; Bertness & Coverdale, 2014; Coverdale et al., 2013). Such findings add complexity to the growing consensus that top‐down consumer control is common in coastal wetlands and suggests that the effect of these factors may instead be highly context‐dependent.

**TABLE 5:** Observed impact of predator presence or absence on measured variables. The positive symbol indicates an increase in the measured variable relative to control conditions while the negative symbol indicates a decrease in the measured variable relative to control conditions. Cells with a single value represent results across all sites, while cells with three values indicate site‐specific outcomes in the following order: Farm River State Park, Fence Creek, and Hammonasset Beach State Park

	Burrow Density	Soil Organic Matter	Nitrogen Content	Nitrogen Absorption	Aboveground Biomass
Predator	0	0	0	0	0
No Predator	‐ / 0 / 0	0	0 / + / 0	0 / 0 / ‐	0 / + / 0

### 4.2 Predator effects

With all study sites combined, predator impacts on prey, as measured by burrow density, did not drive differences across any of the measured ecosystem response variables. Within salt marsh ecosystems, burrow density is a metric commonly used as a proxy for crab prey abundance given the difficulty of determining population sizes of burrowing species by sampling individuals directly (Coverdale et al., 2014; Gittman & Keller, 2013). Burrow density was predicted to decline in the presence of predators due to a reduction in the functional density of burrowing prey. However here, burrow density decreased in the Predator Absent treatment compared with the control, driven by changes at one field site: Farm River State Park. This contradictory outcome may be the result of reduced evasive burrowing activity (a behavioral rather than density effect of predator presence/absence) by fiddler crabs and purple marsh crabs in the absence of a predator (Altieri et al., 2009; Coverdale et al., 2013; Hemmi et al., 2005; Matassa and Trussel, 2011; Tomsic et al., 2017; Trussel et al., 2017; Wong et al., 2005). It may also be that burrow structures that collapsed during the experimental period masked the effect of predation on burrowing prey functional densities (Coverdale et al., 2014). These results, in tandem with the absence of experimental treatment effects on burrow density at the remaining field sites, suggest that observed changes in other response variables at those sites may be due to factors other than changes in prey functional density.

Although predator effects did not influence response variables overall, an interaction between site and experimental treatment indicates context‐dependent changes across several response variables. At Fence Creek, soil nitrogen concentration increased significantly in the Predator Absent and Predator Present treatments compared with the control, which is inconsistent with stated predictions and the burrow density data at this site. Soil nitrogen content is often influenced by burrowing behavior and bioturbation via an increase in soil drainage and oxygenation (Moore, 2019; Wang et al., 2010); soil nitrogen is therefore predicted to be higher in the absence of a predator due to an increase in prey functional density and the concomitant increase in burrow activity. In the absence of explanatory bioturbation, it may be that crabs present in experimental cages facilitated an increase in soil nitrogen concentration via direct inputs such as excrement (Smith et al., 2009).

At this site, there was also significantly more aboveground biomass in the Predator Absent treatment compared with the Predator Present treatment (*p* =.038) with no difference between the experimental treatments and the control (Table 2, Figure 1). These results were driven by changes observed in 2015, where the Predator Absent treatment had significantly more biomass than both the control and Predator Present treatment whereas in 2016, there were no significant differences between treatments. Previous studies have shown that the exclusion of predators should allow herbivorous prey to freely consume vegetation, causing a reduction in standing biomass in these areas relative to those where predators maintain access (Bertness, Brisson, Coverdale, et al., 2014). The results presented here do not follow these expected outcomes, with four potential explanations. (a) Across field sites, fiddler crabs far outnumber purple marsh crabs and variation in fiddler crab density or behavior may swamp out predation effects on herbivorous purple marsh crabs (Wang et al., 2010). If fiddler crabs are being influenced by predators more strongly than purple marsh crabs, then this may manifest as an increase in the rate of soil nitrogen absorption and associated aboveground biomass growth in the absence of predators as indicated by these results. (b) The rate of plant aboveground biomass production may have increased over the course of the experiment to compensate for losses caused by herbivory (Gittman & Keller, 2013). (c) In areas with predators present, herbivory was predominantly belowground which may lead to reduced aboveground growth (Coverdale et al., 2012; Vu & Pennings, 2018). (d) It may take a longer period than was observed in this experimental study for predator effects to cascade through the trophic levels and significantly impact aboveground biomass (Vu & Pennings, 2018). Additionally, although the European green crab is a voracious predator, it has also been known to consume vegetation (Leignel et al., 2014). It may be the case that green crabs directly consumed vegetation within the Predator Present treatment plots, leading to an overall reduction in biomass relative to the plots where green crabs were not present.

At Hammonasset Beach State Park, the rate of soil nitrogen absorption was significantly lower in the Predator Absent treatment compared with the control and marginally lower compared with the Predator Present treatment (Table 3, Figure 2). However, as noted earlier, the use of burrow density as a metrics has limitations that include the possibility that burrow structures collapsed prior to being measured. Therefore, it may be that an increase in burrowing to avoid predation in the Predator Present treatment led to a concomitant increase in the rate of soil nitrogen absorption. This suggests that contrary to initial expectations, Hammonasset Beach State Park likely has a significant predator population; removing this predator led to a concomitant change in the rate of nitrogen absorption while treatments with predators present maintained nitrogen absorption rates similar to those observed in the control treatment.

Path analyses indicate that neither top‐down nor bottom‐up factors strongly influenced changes in measured variables overall, but instead differentially influenced several ecosystem properties across field sites. Though few studies have empirically evaluated the relative influence of bottom‐up and top‐down control in coastal wetlands, those that have highlighted the presence of synergistic effects may be dependent on environmental context (Deegan et al., 2007; Sala et al., 2008). In their evaluation of top‐down and bottom‐up control in a New England salt marsh, Sala et al. (2008) found that bottom‐up factors maintained ecosystem functions under low soil nutrient levels but a combination of bottom‐up and top‐down control was observed under high nutrient levels. Here, the predominance of bottom‐up and top‐down control varied by site: Farm River State Park variables were influenced by top‐down factors while Fence Creek and Hammonasset Beach State Park variables were influenced by a combination of bottom‐up and top‐down factors (Figure 3a‐c). Although these field sites were chosen for their ecological and physical similarities, there are notable differences in their surrounding contexts that may contribute to the differential bottom‐up and top‐down effects observed here. In particular, Farm River State Park is situated in a region along the Connecticut coastline that is heavily residential with significant marine recreational uses nearby. This field site is also downstream from a local farm that may contribute nutrient inputs into the watershed that eventually passes through the site. Although our results do not show a higher soil nitrogen concentration at this site relative to the others, we did observe a significant increase in the rate of soil nitrogen absorption at Farm River which may be due to an influx in soil nutrients that are being rapidly utilized by the vegetation. This local context in tandem with the reduction in SOM content at this site suggests that there may be higher nutrient levels here that facilitate stronger top‐down effects relative to Fence Creek and Hammonasset Beach State Park.

Altogether, the mixed‐effects model and path analysis results shown here are consistent with other studies highlighting variable bottom‐up or top‐down control in various environmental contexts, rather than those championing clear and consistent top‐down or bottom‐up control. Moreover, given our level of experimental replication is consistent with that of other studies detecting strong top‐down control, we suggest that our findings are not a consequence of an inability to detect strong top‐down control due to inadequate replication.

## 5 CONCLUSION

Despite a growing body of literature indicating the importance of consumers and trophic interactions in maintaining ecosystem functions within coastal wetlands, our study presents results that temper these conclusions. Predators did not influence any ecosystem processes when all three experimental sites were combined but instead, differentially influenced variables at each site. Most notably, the absence of predators at two sites facilitated changes in various response variables relative to the control treatment that were not observed in the presence of predators. Such findings highlight a potential context‐dependent role for predators in land management or restoration practices aiming to recover these ecosystem properties. Altogether, the results presented here indicate that predator effects in coastal wetland ecosystems are far from ubiquitous. Additional empirical studies evaluating how and why predator effects vary under different environmental contexts are needed so that we may better understand the ecology of these dynamic ecosystems and improve restoration and conservation outcomes.

## CONFLICT OF INTEREST

To the best of our knowledge, there is no conflict of interest.

## AUTHOR CONTRIBUTION

**Alexandria C Moore:** Conceptualization (equal); Data curation (lead); Formal analysis (lead); Funding acquisition (lead); Investigation (lead); Methodology (equal); Writing‐original draft (lead); Writing‐review & editing (equal). **Oswald Schmitz:** Conceptualization (equal); Formal analysis (supporting); Methodology (equal); Supervision (lead); Writing‐review & editing (equal).

## Data Availability

The full dataset and statistical analysis code for this study are available at: https://doi.org/10.5281/zenodo.5019317.
